# Tree Sapling Responses to 10 Years of Experimental Manipulation of Temperature, Nutrient Availability, and Shrub Cover at the Pyrenean Treeline

**DOI:** 10.3389/fpls.2018.01871

**Published:** 2019-01-08

**Authors:** Maria A. Angulo, Josep M. Ninot, Josep Peñuelas, Johannes H. C. Cornelissen, Oriol Grau

**Affiliations:** ^1^Global Ecology Unit, CSIC, CREAF-CSIC-UAB, Cerdanyola del Vallès, Spain; ^2^Centre de Recerca Ecològica i Aplicacions Forestals, Cerdanyola del Vallès, Spain; ^3^Department of Evolutionary Biology, Ecology and Environmental Sciences, Institute for Research on Biodiversity (IRBio), University of Barcelona, Barcelona, Spain; ^4^Systems Ecology, Department of Ecological Science, Faculty of Earth and Life Sciences, Vrije Universiteit Amsterdam, Amsterdam, Netherlands

**Keywords:** chemical composition, competition, facilitation, fertilization, open-top chamber, *Pinus uncinata* seedlings, Pyrenees, *Rhododendron ferrugineum*

## Abstract

Treelines are sensitive to environmental changes, but few studies provide a mechanistic approach to understand treeline dynamics based on field experiments. The aim of this study was to determine how changes in the abiotic and/or biotic conditions associated with global change affect the performance of tree seedlings (later saplings) at the treeline in a 10-year experiment. A fully factorial experiment in the Central Pyrenees was initiated in autumn 2006; 192 *Pinus uncinata* seedlings were transplanted into microplots with contrasting environmental conditions of (1) increased vs. ambient temperature, (2) increased nutrient availability vs. no increase, and (3) presence vs. absence of the dominant shrub *Rhododendron ferrugineum*. We assessed the performance of young pines on several occasions over 10 years. The pines were removed at the end of the experiment in autumn 2016 to characterize their morphology and to conduct chemical and isotopic analyses on their needles. Both the warming and the fertilization treatments increased seedling growth soon after the start of the experiment. *R. ferrugineum* facilitated the survival and development of pine seedlings during the early years and affected the chemical composition of the needles. Toward the end of the experiment, the transplanted *P. uncinata* individuals, by then saplings, competed with *R. ferrugineum* for light and nutrients; the presence of the shrub probably altered the strategy of *P. uncinata* for acquiring nutrients and buffered the effects of warming and fertilization. The pines were highly sensitive to all factors and their interactions throughout the entire experimental period. These findings indicated that the interactive effects of several key abiotic and biotic drivers associated with global change should be investigated simultaneously for understanding the contribution of young trees to treeline dynamics.

## Introduction

Treeline ecotones are highly sensitive to climatic warming, because air and soil temperatures limit growth at high elevations and latitudes, where the growing season is generally short (Holtmeier, 2009; Körner, 2012). Many studies during the last decade have focused on the potential shifts in the position of treelines in response to climate change that leads to warmer temperatures (Grace et al., 2002; Körner and Paulsen, 2004; Moen et al., 2004; Peñuelas et al., 2007; Harsch et al., 2009; Barbeito et al., 2012; Yadava et al., 2017). Climatic warming, however, is only one aspect amongst several that control the altitudinal or latitudinal movements of treelines (Holtmeier and Broll, 2005; Lyu et al., 2016). Other factors may also shape treeline dynamics: abiotic factors such as wind velocity, solar radiation, and duration of snow cover (Wipf et al., 2009); and increases in nitrogen (N) deposition (Holtmeier and Broll, 2005), atmospheric CO_2_ concentration (Hättenschwiler and Zumbrunn, 2002; Handa et al., 2006), and ozone concentration (Díaz-de-Quijano et al., 2012; Huttunen and Manninen, 2013); but also biotic factors such as plant–plant interactions (facilitation or competition for abiotic resources) (Germino et al., 2002; Grau et al., 2012; Liang et al., 2016; Lyu et al., 2016), dispersal patterns (Vetaas and Grytnes, 2002), damage caused by herbivory (Munier et al., 2010), and changes in land use (Hofgaard, 1997). Many of these factors generally operate simultaneously and may interact, so providing clear mechanistic explanations for shifts in treelines based on reproducible experiments is difficult. The underlying factors that cause treeline shifts are thus not yet fully understood.

Marked regional displacements of treelines to higher altitudes or latitudes have occurred in the past. For example, treelines migrated upwards during a warm period in the early Holocene in many northern regions, such as the Scandinavian Mountains (Kullman, 1999), northern Eurasia (MacDonald et al., 2000), and central and western Canada (Spear, 1993). No general patterns, however, have been observed at the continental scale during the last century. In fact, a global meta-analysis (Harsch et al., 2009) reported that 87 of 166 treelines had advanced, 77 were stable, and two had receded since 1900, suggesting that the upward displacement of treelines is not a general world-wide phenomenon. Liang et al. (2011) reported that a treeline on the Tibetan Plateau had become denser because of an increase in the number of seedlings, but it had not moved significantly upslope. Gehrig-Fasel et al. (2007) reported that 10% of the treelines studied in the Swiss Alps had shifted upwards between 1985 and 1997 and that the woody vegetation in the other 90% had become denser. A regional densification of treeline vegetation was detected in the Catalan Pyrenees and Andorra during the second half of the 20th century (Batllori and Gutiérrez, 2008). Some Pyrenean treelines, though, shifted upwards by almost 40 m between 1956 and 2006, especially those where the cessation of human activity (livestock grazing, fire, logging) was more evident, whereas some other treelines in this region have responded little or not at all in recent decades (Ameztegui et al., 2015; Camarero et al., 2015). Identifying general patterns over large regions is thus difficult. Here we postulate that this lack of common response is due to other abiotic and biotic drivers influencing the response of trees to temperature at or near the treeline.

The cover of shrubs and the ‘shrubline’ may also vary along with the changes in tree density or altitudinal/latitudinal shifts observed in some treelines (Hallinger et al., 2010). These changes are relevant because shrubs are potential modifiers of abiotic conditions at the microhabitat scale (Myers-Smith et al., 2011), so the expansion of shrubs across the treeline may play a role in treeline dynamics (Liang et al., 2011; Grau et al., 2013). For example, an increase in shrub cover favors the accumulation of snow leeward of the shrubs (Sturm and Holmgren, 2001; Wipf et al., 2009), thereby protecting tree seedlings from damage caused by low temperatures and snow abrasion (Germino and Smith, 1999; Holtmeier and Broll, 2007). Shrubs can also protect tree seedlings from strong winds or high irradiance during the growing season, which can affect their performance and photosynthetic rates (Akhalkatsi et al., 2006; Batllori et al., 2009; Grau et al., 2013). These tree seedlings, however, do not necessarily form a treeline over time, because most individuals will die or become ‘Krumholz’ trees, despite the facilitative effects of shrubs (Ninot et al., 2008). Furthermore, trees and shrubs may compete for resources such as light, nutrients, or water during later stages of development, so initial facilitation may not necessarily lead to the development of mature trees at the treeline (Wang et al., 2016). This idea was reinforced by Barbeito et al. (2012), who observed that shrubs could inhibit the development of trees in the Swiss Alps. Recent changes in shrub cover may nevertheless have a greater impact on treeline dynamics than recent changes in temperature (Dullinger et al., 2004; Liang et al., 2016); more research is needed to understand the impact of increases in shrub cover on treeline shifts and the interaction between changes in shrub cover and abiotic regimes.

Many of the studies conducted in treeline ecotones have described the observed patterns (e.g., treeline shifts, densification, and stability), but only few studies have analyzed these patterns experimentally. A few studies have investigated the effects of climatic warming on the performance of tree seedlings or samplings at the treeline. Munier et al. (2010) concluded that climatic warming would displace treelines upwards only if viable seeds and suitable substrates were available. Nutrient (especially N) availability at treelines is generally low, because low soil temperatures limit the rates of microbial decomposition and mineralization and nutrient uptake (Chapin, 1983; Birmann and Körner, 2009; Mcnown and Sullivan, 2013). An increase in soil temperatures, however, is expected to increase nutrient availability (Hobbie, 1996) and tree productivity at the treeline (Mack et al., 2004). Sveinbjörnsson et al. (1992) found that the establishment of *Betula pubescens* at a Swedish treeline was favored under increased N availability, and Hoch (2013) found that fertilization doubled the productivity of *Larix decidua* and *Pinus uncinata* when temperature was experimentally increased. It remains unknown, however, how important nutrient limitation is for tree performance at the treeline, and whether the increase of nutrient availability through potential increases in mineralization will compensate the nutritional demands of trees that grow under warmer conditions.

Few studies, have investigated the interactions amongst several of the factors that control the performance of trees at the treeline, such as temperature, nutrient availability, and shrub cover. Experimental studies where all these factors are combined are essential to find out whether nutrient limitation or shrub cover could alter or buffer the effects of temperature. Grau et al. (2012) conducted a multifactorial experiment in Swedish Lapland where *Betula pubescens* seedlings grew under contrasting environmental scenarios involving a full factorial combination of presence vs. absence of the shrub *Vaccinium myrtillus*, increased vs. ambient warming, and increased nutrient availability vs. no increase. This study found that treeline dynamics were driven by complex environmental interactions amongst these factors and that facilitation, competition, herbivory, and environmental changes at the tree seedling stage acted as important filters in structuring the treeline ecotone. Another experiment with the same factorial design was conducted in a more southern treeline, in the Pyrenees (Grau et al., 2013). In this region, mean annual temperatures increased (Cuadrat et al., 2013; Martín-Vide et al., 2017), snow cover decreased (López-Moreno, 2005; Cuadrat et al., 2013; Martín-Vide et al., 2017), and shrub cover expanded over the last decades (Molinillo et al., 1997; Roura-Pascual et al., 2005; Montané et al., 2007; Alados et al., 2011; Ninot et al., 2011; Garcia-Pausas et al., 2017). The tree and shrub used in this experiment, though, were *P. uncinata* and *Rhododendron ferrugineum*, which are dominant across the Pyrenean treeline. The seedlings of *P. uncinata*, which commonly forms the treeline in the Central Pyrenees, were highly sensitive to the simulated environmental changes within 3 years after transplantation (see Grau et al., 2013 for further details). The seedling stage is crucial but is only a short phase in the life of a treeline tree. Saplings, for example, being taller, are likely exposed to different abiotic and biotic environments than seedlings (Körner, 1998; Chrimes, 2004). Here we argue that understanding tree performance and associated treeline dynamics requires determining the complex interactions of abiotic and biotic drivers over time as the trees grow taller.

We re-visited the upper treeline site established in 2006 by Grau et al. (2013) in the Pyrenees 10 years after the onset of the experiment. We assessed the contributions of multiple interactive drivers of tree performance through time and whether the responses of the tree seedlings during the early years after transplantation persisted or varied over time. To our knowledge, this treeline experiment is the first and longest to test the responses of trees to contrasting environmental scenarios involving abiotic and biotic drivers simultaneously. In the initial study (Grau et al., 2013), the *P. uncinata* seedlings responded positively to the presence of *R. ferrugineum* shrubs, which provided protection to seedlings against winter damage. Both higher temperatures and increased nutrient availability had positive effects on seedling development. The positive effects of warming, however, were more marked in the absence of the shrub. In agreement with the observations in the initial study, we hypothesized that tree saplings in the Pyrenean treeline 10 years after transplantation would grow better (better development and higher foliar nutrient content) (1) under the protection of the shrubs, (2) in plots with increased temperatures, and (3) in plots with increased nutrient availability. We also hypothesized that some factors would interact (4) negatively (e.g., presence of the shrub and warming) or (5) positively (e.g., warming and nutrient addition). We tested these hypotheses to improve our knowledge of the factors that control longer term treeline dynamics in the Pyrenees and to provide a robust mechanistically based framework for extrapolating the impacts of environmental changes on treeline dynamics to other regions.

## Materials and Methods

### Study Area

The experiment was conducted on the north-western slope of Serrat de Capifonts (Pallars Sobirà, 42°33′N, 01°23′E) in Alt Pirineu Natural Park (Central Pyrenees, Catalonia, Spain) (Supplementary Figure S1). The experimental area was located in the upper part of the treeline at 2400 m a.s.l. (Carreras et al., 1996), dominated by scattered *P. uncinata* individuals (generally <2 m high) and *R. ferrugineum* shrubs, with patches of grassland that become progressively dominant above the treeline. The macroclimate of this part of the Pyrenees is montane continental due to its intermediate position between the Mediterranean Sea and the Atlantic Ocean (del Barrio et al., 1990). The 0°C isotherm from November to April is at about 1600–1700 m a.s.l., which indicates the lower limit where snow accumulates for a long period (López-Moreno et al., 2009). The growing season is normally about 5 months at the treeline; it generally starts when the air temperature remains above 5°C for more than five consecutive days and ends when temperatures are lower than 5°C for several days (Grau et al., 2013). The nearby meteorological station in Salòria (42°31′N, 01°21′E; 2451 m a.s.l.) recorded a mean annual temperature of 2.45 ± 6.77°C and a mean annual precipitation of 946 ± 315 mm between 2006 and 2015. The mean maximum daily air temperature in summer (June–August) was 13.43 ± 4.08°C, and the mean minimum daily air temperature in winter (December–February) was -3.6 ± 4.77°C. The snow cover lasted 160 ± 50.4 days, although the winters in 2005–2006 and 2006–2007 were exceptionally dry and the snow lasted for only about 100 days. The maximum snow depth indicated great interannual variability, with mean depths generally exceeding 1 m in winter (Supplementary Figure S2).

### Study Species

We transplanted *P. uncinata* seedlings in this experiment because this tree species forms most of the altitudinal treelines on the southern slopes of the Central Pyrenees. This pine species is a long-lived, slow-growing, and shade-intolerant conifer with a wide ecological tolerance for topography (slope, exposure, and elevation) and soil type, because it is highly tolerant to stress (Batllori et al., 2010). *R. ferrugineum* is one of the most abundant shrubs in subalpine environments at elevations around 1600–2200 m a.s.l. (Pornon et al., 2000). It usually covers the understory of subalpine forests up to the treeline and may form scrub upslope, toward the alpine belt. It appears in places where the snow accumulates for an extended period, so it remains protected from low temperatures. It is an ericaceous evergreen shrub mostly on siliceous soils, and its stems may reach a height of approximately 50 cm at the treeline.

### Experimental Design

The *P. uncinata* seedlings had been grown in a nursery from seeds collected in the Central Pyrenees. The seedlings were transplanted in the experimental site in autumn 2006 when they were 8–10 cm tall. We focused on the performance of young trees in this experiment because the early developmental stages are highly sensitive and responsive to environmental changes, thereby strongly determining future treeline dynamics (Barbeito et al., 2012).

The fully factorial experimental design included three factors that simulated contrasting environmental scenarios, with a total of eight treatments and four replicates per treatment. These three binary factors, assumed to be the most critical to the performance of pine seedlings, were: (1) increased temperature (+T) vs. ambient temperature (-T), (2) increased nutrient availability (+F) vs. no increase (-F), and (3) presence (+S) vs. absence (-S) of the dominant shrub *R. ferrugineum* (Supplementary Figure S3). These treatments were distributed randomly over 16 experimental units of the same area (1.32 m^2^) (Supplementary Figure S4). We had a total of four experimental units (used as replicates) for the treatments, +T, +F, and +T+F, and four -T-F experimental units that were used as controls. Each of these treatments was combined with the presence (+S) or absence (-S) of the shrub, and each experimental unit was placed at the edge of a *R. ferrugineum* patch (approximately 50 cm tall), so that half of the experimental unit was covered by the shrub and the other half was not (two microplots per experimental unit). Six seedlings were transplanted into each microplot, for a total of 192 seedlings (16 experimental units × 2 microplots × 6 seedlings). The vegetation growing inside the microplots without the shrub was regularly cut aboveground to avoid any re-growth. The effect of temperature was simulated with passive warming with OTCs of 1.32 m^2^ in area and 50 cm high, designed based on the hexagonal ITEX model (Marion et al., 1997). Toward the end of the experiment, the upper part of the stems of the saplings in the -S+T+F and the -S+T-F treatments were already taller than the OTC. Although most of the stem was still inside the chamber, we decided to finalize the experiment at this point to avoid that the upper parts of the saplings in these treatments would become exposed to different temperatures than the lower parts. The temperature inside the OTCs was monitored by temperature loggers during the first 4 years of the experiment (iButtons 1-wire Thermochron temperature logger, Dallas Semiconductor Corporation, Dallas, United States). The temperature at ground level was approximately 2°C higher in the warmed experimental units than the control units during the growing season and this increase was consistently detected through the monitoring period. The aboveground plant tissues inside the OTC, though, may have been exposed to higher temperatures than at ground level due to wind speed reduction inside the chamber, and the sensitivity of the saplings to warming was therefore possibly overestimated (De Boeck et al., 2012). The units that did not have an OTC were also hexagonally shaped to maintain the uniformity of all experimental units. The OTCs were removed every winter to avoid different patterns of snow accumulation amongst the experimental units, so we did not account for any advance of the snowmelt in spring. We replaced the OTCs soon after the snow had melted to simulate temperature changes only over the growing season. Finally, fertilizer was applied to the ground surface to simulate an increase in the mineralization rate and thus in the availability of nutrients (Rustad et al., 2001). The nutrients were added only once, in June 2007, by adding 200 g of slow-release NPK (10% N, 5% P_2_O_5_, 20% K) granules in each fertilized microplot.

### Data Collection and Laboratory Analyses

Stem height was measured when the seedlings were transplanted in autumn 2006 and yearly from 2007 to 2009 at the end of the growing season (end of September or early October). Stem height was measured again 10 years later, in June and October 2016. We assumed that the heights measured in June 2016, before the onset of the growing season, corresponded to the growth until 2015. We also assessed survival in each survey, and on some occasions we also measured the stem diameter and the number of branches.

The pine saplings were removed from the ground in October 2016 and transported to the laboratory in plastic bags. The roots were cut off, and we measured several morphometric variables: stem height (including only the woody stem without the upper needles), number of primary branches, basal diameter, and number of branches that grew during the last growing season. All saplings were then oven-dried at 65°C to a constant weight (usually 72 h), and total aboveground biomass, biomass of needles grown in 2016 (new needles), and biomass of new stems grown in 2016 were measured.

A few needles from each sapling were collected from each microplot and pooled into a composite sample (a minimum of 2 g per sample, with an equal weight of needles from each sapling in a given microplot). The needles were ground with a MM400 of Retsch (Haan, Germany) and stored in Eppendorf tubes. These samples were used for the analysis of the elemental and isotopic composition of the pine needles. The macro- and microelements were analyzed to determine whether the experimental treatments affected the nutritional status of the trees, their sources of N uptake (∂N^15^), or the water-use efficiency (∂C^13^). We also tested the ‘biogeochemical niche hypothesis’, which predicts that changes in abiotic and biotic conditions will alter the stoichiometric composition of plant tissues (Peñuelas et al., 2008; Urbina et al., 2017), by analyzing a wide spectrum of chemical elements. The foliar concentrations of Na, Mg, P, S, K, Ca, V, Cr, Mn, Fe, Ni, Cu, As, Sr, Mo, Cd, and Pb were estimated from digested dilutions using inductively coupled plasma mass spectrometry in the laboratories of the Universitat Autònoma de Barcelona. The concentrations of C, N, ∂N^15^, and ∂C^13^ were analyzed by depositing 3.5 mg of dried and ground sample in aluminum capsules, which were sent to a laboratory at the University of California, Davis (UC Davis Stable Isotope Facility). The samples were run on an Elementar Vario EL Cube elemental analyzer (Elementar Analysen systeme GmbH, Hanau, Germany) connected to a PDZ Europa 20-20 isotope ratio mass spectrometer (Sercon Ltd., Cheshire, United Kingdom).

### Statistical Analyses

The effect of each treatment on each variable for each year was analyzed with a linear mixed model as implemented in R v. 3.3.2 (R Core Team, 2017), using the ‘nlme’ (Pinheiro et al., 2017) and ‘lme4’ (Bates et al., 2015) packages. ‘Microplots’ and ‘shrubs’ (nested within ‘microplots’) were considered as random factors to account for the grouping structure of the data. The interaction terms and factors for a given variable that had no statistical support (*p* > 0.05) were removed from the model. The significance of the remaining factors and interactions was recalculated every time a term was excluded from the analyses, provided that the new model was an improvement (*p* < 0.05) over the more complex model in a likelihood ratio test. The significance of each factor was based on the minimally adequate model. We also conducted pairwise tests with Bonferroni correction (Bland and Altman, 1995) for comparing the effects of the presence vs. absence of the shrub on *P. uncinata* growth for the +T, +F, and +T+F treatments.

In addition, a ‘repeated-measures analysis’ was conducted to determine the influence of time on stem height. The model used in this analysis also included an autocorrelated error term that took into account the repeated measures on the same individuals throughout the experimental period. We compared the models that included the temporal term with the models that did not and chose the minimally adequate model with the lowest Akaike’s information criterion (Akaike, 2011).

We conducted principal component analyses (PCAs) with the R ‘FactoMineR’ package to determine the differences between the trees growing in control plots vs. those in the other treatments based on the measured variables (tree morphometry and foliar nutrient concentration).

## Results

**FIGURE 1:**
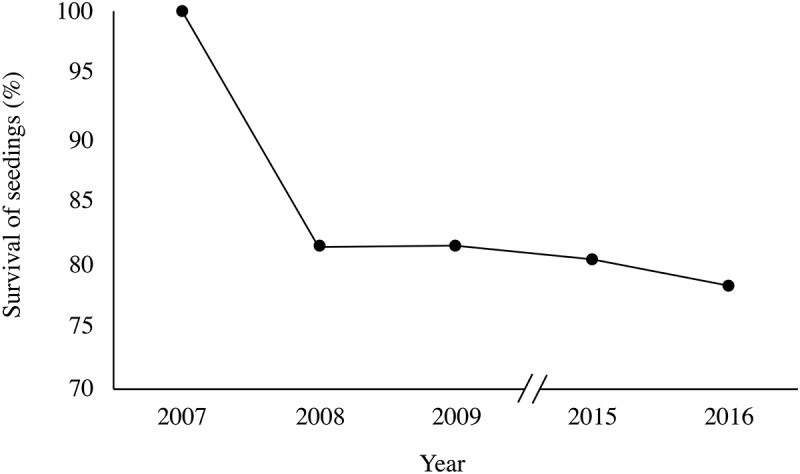
Survival of the transplanted *P. uncinata* individuals over the course of the experimental period.

**FIGURE 2:**
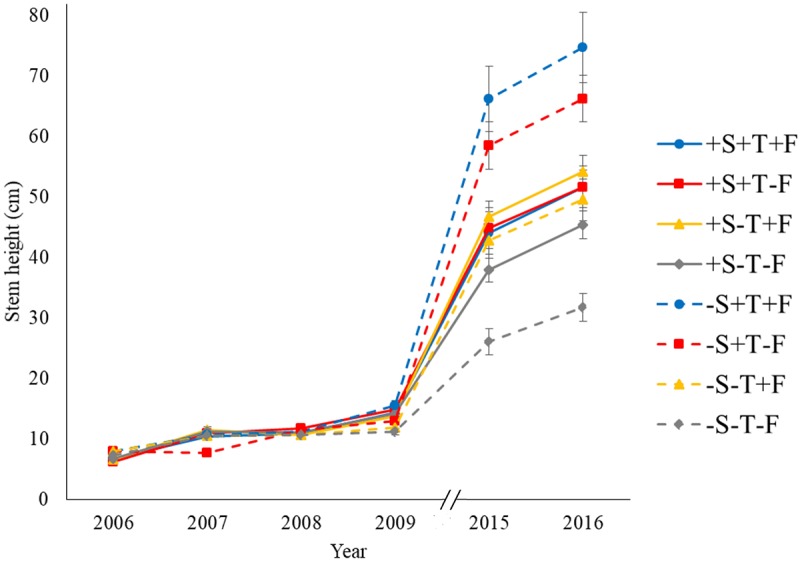
Mean stem height over the course of the experimental period. Treatments: –S, without the shrub; +S, with the shrub; –T, without OTC; +T, with OTC; –F, without fertilizer; +F, with fertilizer.

**FIGURE 3:**
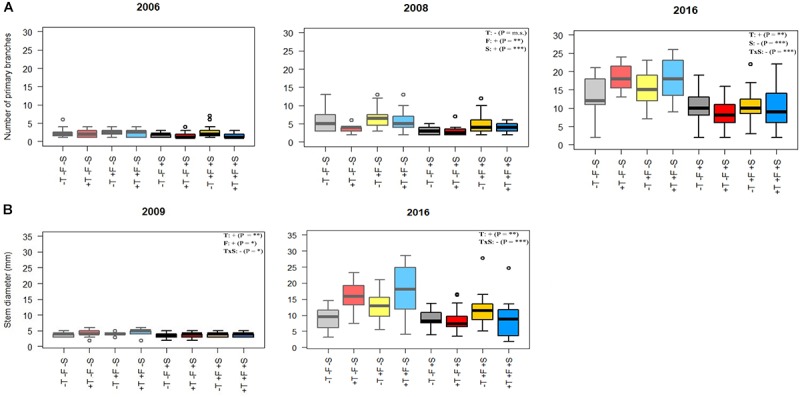
**(A)** Number of primary branches and **(B)** stem diameter in various sampling periods. Light colors (left half of each plot) represent –S treatments, and darker colors (right half of each plot) represent +S treatments. Gray, control treatment (–T–F); red, warming (+T); yellow, fertilization (+F); blue, warming and fertilization (+T+F). See Supplementary Tables S3, S4 for the statistical significance of each factor. ^∗^*p* < 0.05; ^∗∗^*p* ≤ 0.01; ^∗∗∗^*p* ≤ 0.001; m.s., marginally significant.

Seventy-eight percent of the 192 transplanted seedlings survived until the end of the experiment (Figure 1), but survival did not differ between treatments. Warming and the presence of *R. ferrugineum* influenced most of the other variables measured in the transplanted trees, and most of their effects increased over the course of the experiment. Fertilization had a mostly positive effect (Figures 2, 3 and Supplementary Table S1). The means of the morphometric and chemical variables are presented in Supplementary Tables S2–S6.

### Effects of the Experimental Conditions on Sapling Growth and Biomass

**FIGURE 4:**
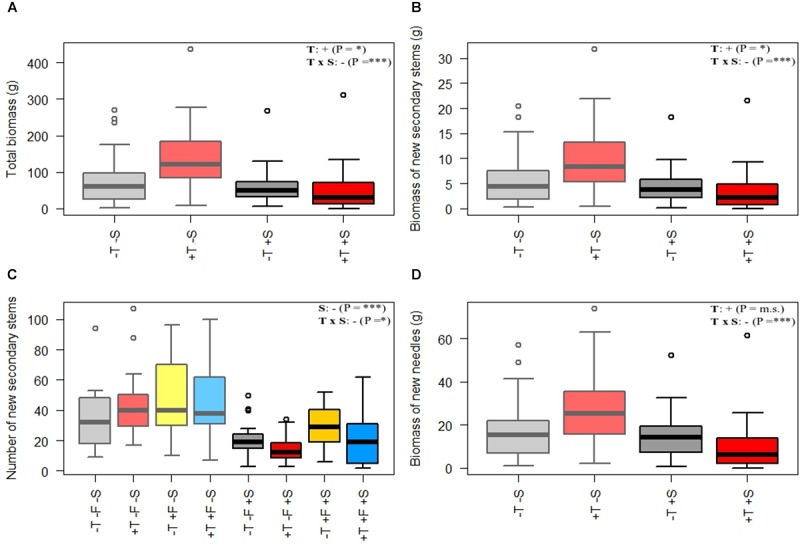
Morphological variables measured in autumn 2016 for each treatment: **(A)** total biomass, **(B)** biomass of new secondary stems, **(C)** number of new secondary stems, and **(D)** biomass of new needles. The statistical differences between treatments are shown in Supplementary Table S7. Light colors (left half of each plot) represent –S treatments, and darker colors (right half of each plot) represent +S treatments. Gray, control treatment (–T–F); red, warming (+T); yellow, fertilization (+F); blue, warming and fertilization (+T+F). See Supplementary Table S5 for the statistical significance of each factor. ^∗∗^*p* ≤ 0.01; ^∗∗∗^*p* ≤ 0.001; m.s., marginally significant.

Experimental warming had a strong positive effect on stem height (Figure 2) and diameter over time (Supplementary Tables S1A,B). The number of primary branches was also positively affected by warming at the end of the experiment (Figure 3A and Supplementary Table S1C). Warming did not significantly increase the number of new secondary stems, but their biomass and the biomass of new needles increased (Figure 4 and Supplementary Table S7). The warming treatment, however, interacted with the shrub treatment; stem height, basal diameter, the number of primary branches and new secondary stems, the biomass of new needles, and the total biomass increased to a much lesser extent or did not increase in response to warming when *R. ferrugineum* was present.

The addition of fertilizer also increased stem growth over the course of the experimental period (Figure 2 and Supplementary Table S1A) although it interacted with the presence of *R. ferrugineum*, as observed in the warming treatment. The effect of the fertilizer tended to be more evident in 2009, 2015, and 2016 if the shrub was not present. Branching increased significantly only in autumn 2008 (Figure 3A), and stem diameter increased significantly only in 2009 (Figure 3B).

**FIGURE 5:**
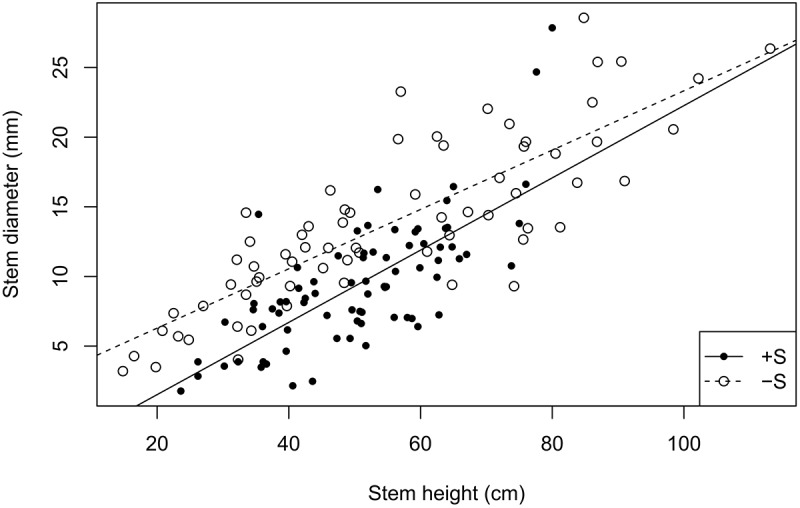
Regression between stem height and basal diameter with and without the presence of *R. ferrugineum*. The intercepts of the +S and –S treatments are –3.67 and 2.03, respectively, and the *R*^2^ 0.522 (*P* = <0.0001) and 0.682 (*P* = <0.0001).

Stem height did not increase significantly in the presence of *R. ferrugineum* in the repeated-measures analyses when the effect of the shrub alone was analyzed. The stems in 2009, 2015, and 2016, however, were significantly longer in the presence of *R. ferrugineum* when each year was analyzed separately (Figure 2 and Supplementary Table S1A). *P. uncinata* saplings growing with the shrub tended to have thinner stems, although this trend was not significant, and their biomass did not vary (Figures 3B, 4A and Supplementary Tables S1B, S7). Trees growing without *R. ferrugineum* developed thicker stems than those growing in the presence of the shrub while stems were short. When stem height was lower than 40 cm, the stem was much thinner in trees growing with the shrub. This difference disappeared, however, as trees became saplings (Figure 5).

**FIGURE 6:**
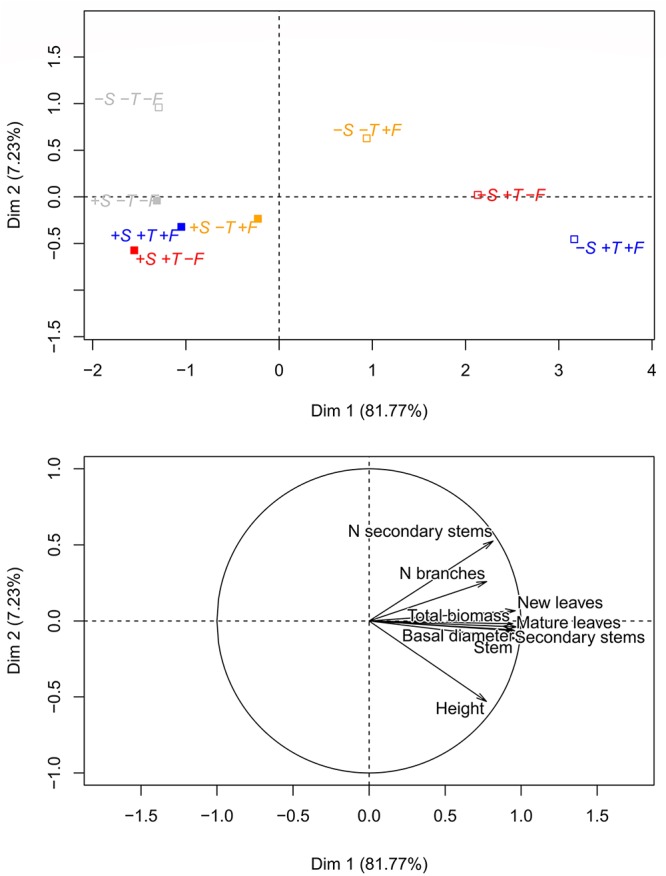
Principal component analyses (PCA) analysis of all morphological variables measured at the end of the experiment, in autumn 2016 (stem height, number of primary branches, number of secondary stems, basal diameter, biomass of secondary stems, biomass of new needles, biomass of old needles, biomass of the stem, and total biomass). The graphic ordination on the two first axes and the centroid of each treatment are shown in the **(upper)** panel, and the direction and strength of the morphometric variables in the PCA are shown in the **(lower)** panel. Open centroids in the upper panel represent the –S treatments, and solid centroids represent the +S treatments. Gray, control treatment (–T–F); red, warming (+T); yellow, fertilization (+F); blue, warming and fertilization (+T+F).

The PCA ordination in Figure 6 illustrates the impact of the contrasting experimental conditions on the morphometric characteristics of the saplings 10 years after the onset of the experiment. Trees growing in altered conditions (+T, +F, and +T+F) without *R. ferrugineum* differed much more from the initial conditions (control plots) than the trees growing with *R. ferrugineum.*

### Effects of the Experimental Conditions on the Chemical Composition of Sapling Needles

None of the treatments significantly affected foliar C or N concentrations 10 years after the fertilizer was applied. The concentrations of P, K, and Cu increased, and the concentration of Mn marginally decreased. The N:P, C:K, and N:K ratios decreased with fertilization (Supplementary Table S8). Warming significantly increased the concentrations of Mn and Zn and marginally significantly increased the concentrations of K and Cu. The presence of *R. ferrugineum* had a positive effect on K and Zn concentrations but had a negative effect on Mn concentration. The Sr concentration increased marginally when warming was combined with the presence of *R. ferrugineum*.

**FIGURE 7:**
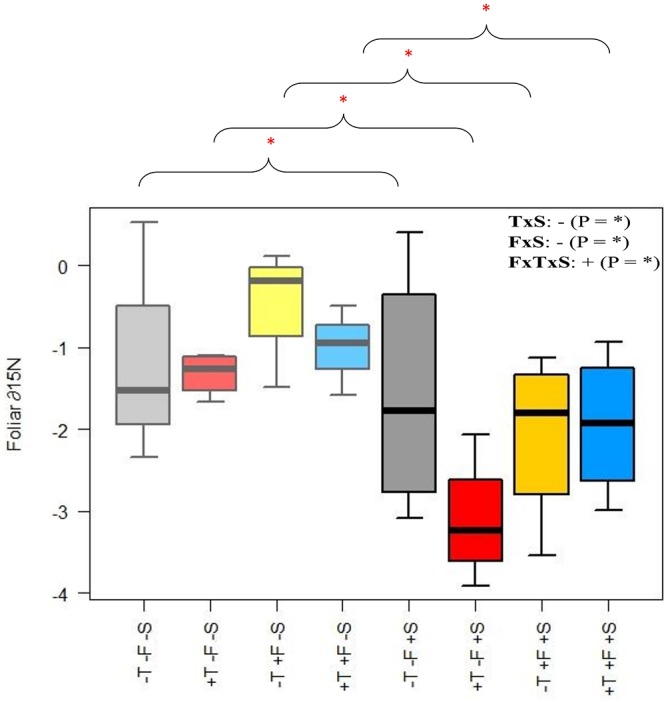
Foliar ∂N^15^ for each treatment. The +S and –S treatments differ significantly for the T, F, and TF treatments for pairwise comparisons of each treatment (asterisks indicate that these paired treatments differ significantly from each other; *p* < 0.05 for each pair). Light colors (left half of each plot) represent the –S treatments, and darker colors (right half of each plot) represent the +S treatments. Gray, control treatment (–T–F); red, warming (+T); yellow, fertilization (+F); blue, warming and fertilization (+T+F). ^∗^*p* < 0.05.

The presence of *R. ferrugineum* did not significantly affect needle ∂N^15^, but ∂N^15^ tended to be lower when the presence of the shrub was combined with warming and/or fertilization (Figure 7 and Supplementary Table S9A). We investigated this trend in more detail by comparing the foliar ∂N^15^ values for the +T, +F, and +T+F treatments with *R. ferrugineum* with the values for the same treatments but without the presence of the shrub using pairwise tests. ∂N^15^ was lower when *R. ferrugineum* was present for each of the +T, +F, and +T+F treatments.

**FIGURE 8:**
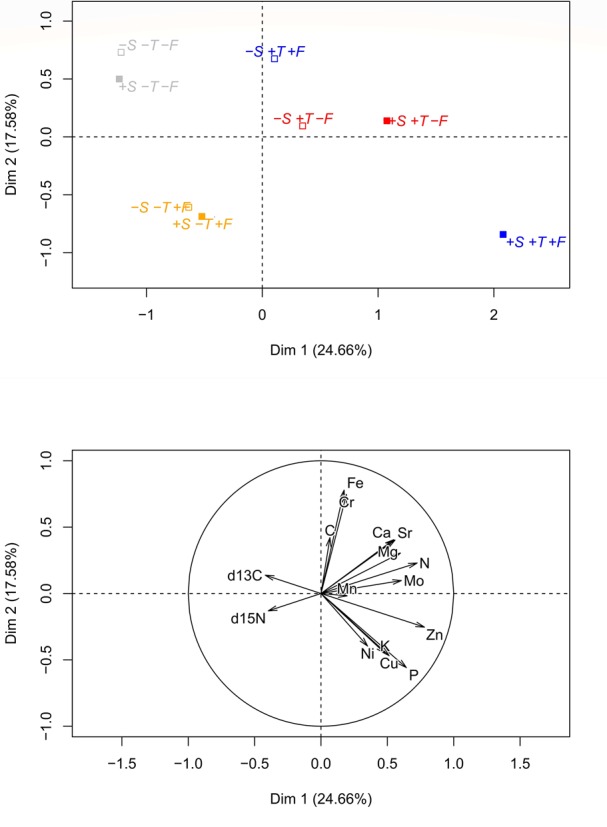
Principal component analyses analysis of the foliar chemical characteristics for each treatment at the end of the experiment, in autumn 2016. The graphic ordination on the two first axes and the centroid of each treatment are shown in the **(upper)** panel, and the direction and strength of the chemical variables in the PCA are shown in the **(lower)** panel. Open centroids in the upper panel represent the –S treatments, and solid centroids represent the +S treatments. Gray, control treatment (–T–F); red, warming (+T); yellow, fertilization (+F); blue, warming and fertilization (+T+F).

The PCA of the foliar chemical composition (Figure 8) indicated that the saplings growing in altered conditions (+T, +F, and +T+F) generally differed greatly from the control saplings, similar to the morphometric variables. The saplings growing in altered conditions (+T, +F, and +T+F) with *R. ferrugineum*, however, tended to differ more from the control saplings than those growing in altered conditions without *R. ferrugineum*, especially for the +T and +T+F treatments.

Warming had a positive effect on the total contents of P, Mn, Cu, and Zn (the product of the concentration of each element and the total needle biomass of the sapling) when *R. ferrugineum* was not present (Supplementary Table S9B). Fertilization only had a marginally positive effect on total Cu content but had no effect on the total contents of the other elements. The contents of some of the elements analyzed (Na, S, V, As, Cd, and Pb) were below the detection limit and were thus excluded from the analyses.

## Discussion

### Biotic and Abiotic Manipulations

In the initial study, Grau et al. (2013) reported that the *P. uncinata* seedlings were facilitated by *R. ferrugineum*. This shrub protected the seedlings from damage in the winter of 2007–2008, when snow cover was exceptionally low during the coldest months (Supplementary Figure S2). The accumulation of snow leeward of the shrubs and the insulation capacity of the snow protected the seedlings from snow abrasion and low temperatures (Smith et al., 2003; Neuner, 2007; Ninot et al., 2008). The presence of *R. ferrugineum* was therefore a crucial factor for seedling development during this early stage of development. We found no further evidence of winter facilitation by shrubs after the winter of 2007–2008, possibly because snow cover was not critically low again until the end of the experiment (Supplementary Figure S3). The potential facilitation by the shrub was nevertheless expected to occur, especially during the initial stages of development when the trees were much shorter than the protective shrubs.

We found evidence, however, that the transplanted trees competed with the shrubs for resources over the course of the experiment. *P. uncinata* individuals tended to develop longer but thinner (i.e., etiolated) stems when grown with *R. ferrugineum*, although their total aboveground biomass did not differ significantly from *P. uncinata* grown without the shrub (Figure 4A and Supplementary Table S7). *P. uncinata* is intolerant of shade (Ninot et al., 2007; Batllori et al., 2010), so this response most likely helped it to adjust to the lack of light when grown in the presence of *R. ferrugineum* (Cranston and Hermanutz, 2013). The trees became progressively less shaded or were no longer shaded when they became taller, and the etiolation tended to disappear (Figure 5). Hypothesis (1), that *P. uncinata* individuals would grow better in the presence of *R. ferrugineum*, was thus only supported for the early stage of life. We observed a sequence over the course of the experiment of a facilitative and then a competitive impact of the shrub, with a final release from competition by the shrub. The competition between *P. uncinata* and *R. ferrugineum* did not significantly affect the concentration of most of the chemical elements of the pine needles. This lack of effect was unexpected, at least for some elements. For example, N is expected to be limiting in cold ecosystems such as treelines (Körner, 2003), where the short growing season and recalcitrancy of plant material limit soil microbial activity and the decomposition of organic matter (Loomis et al., 2006; Macek et al., 2012). Pines, however, nearly always have ectomycorrhizal fungi (Harley and Harley, 1987) and can decompose relatively recalcitrant organic matter, which makes them relatively independent of the availability of inorganic nutrients provided by mineralization or experimental fertilization (Read, 2003). Possibly also indirectly related to mycorrhiza, we observed that the foliar ∂N^15^ of the saplings in the +T, +F, and +T+F treatments was lower with than without *R. ferrugineum* in pairwise comparisons (Figure 7). Foliar ∂N^15^ was also significantly lower in the +S control treatment than in the -S control treatment. Some studies have argued that variations in ∂N^15^ may indicate changes in the strategy of N uptake in plants (Michelsen et al., 1996; Russo et al., 2013). Lower ∂N^15^ generally indicates more N uptake by ectomycorrhizal or ericoid mycorrhizal fungi (Michelsen et al., 1998), with more recycled N leading to lower N losses from the ecosystem (Garten, 1993; Robinson, 2001; Craine et al., 2009; Anadon-Rosell et al., 2016). *R. ferrugineum* has ericoid mycorrhizal fungi (Straker, 1996), so the lower ∂N^15^ values when the shrub was present could indicate that the pines had taken up more N that had been recycled by ericoid mycorrhizal fungi in a relatively closed N cycle. This recycled N with low ∂N^15^ values could still have been mineralized before uptake, but the pines had likely taken up part of their N in organic form derived from *R. ferrugineum* litter, thereby overcoming inorganic-N limitation (Akhmetzhanova et al., 2012). This adaptation may eventually lead to similar foliar N concentrations with or without the presence of *R. ferrugineum*, even though the strategy of N uptake may differ. None of the treatments had any effect on foliar ∂C^13^ (Supplementary Table S6), suggesting that water-use efficiency did not differ significantly between treatments (Sullivan and Sveinbjörnsson, 2011).

Our hypothesis (2), that trees would grow more with warming, was clearly supported. Stem height increased in the warmed plots [approximately twofold more than the control trees when the shrub was not present (Supplementary Table S2)], and basal diameter, number of primary branches, and biomass of the new secondary stems also increased (Figures 2–4 and Supplementary Tables S1, S7). In fact, the *P. uncinata* seedlings were highly sensitive to warming soon after transplantation (Grau et al., 2013), and this sensitivity persisted. Temperature strongly controls photosynthetic rates (Danby and Hik, 2007), root activity (Du and Fang, 2014), meristematic activity and tissue development (Körner, 1998) during the growing season, particularly at the treeline (Körner and Paulsen, 2004). Warming only affected the concentrations of K, Mn, and Zn, but the total contents of P, Mn, Cu, and Zn increased (Supplementary Tables S9A,B). The increase in total content but no change in concentration indicated that *P. uncinata* individuals growing in warmed conditions did not suffer from a dilution of their nutrients and that nutrient acquisition kept pace with the increase in biomass. This finding suggests that warming was generally positive and improved the overall performance of *P. uncinata* individuals.

The addition of fertilizer supported our hypothesis (3), that *P. uncinata* would grow better if nutrient availability increased. The addition of fertilizer enhanced the performance of *P. uncinata* over the course of the experiment (Figures 2, 3B and Supplementary Tables S1A,C), even though the fertilizer was applied only once in 2007.

The effect of warming on *P. uncinata*, however, was significantly lower when *R. ferrugineum* was present (Figures 2–4 and Supplementary Tables S1, S7), supporting hypothesis (4) that warming and the presence of the shrub could interact negatively. When warming was combined with the presence of *R. ferrugineum, P. uncinata* grew significantly less, and foliar nutrient concentration and content did not increase, suggesting that the occurrence of shrubs could strongly buffer or diminish the effects of warming.

The foliar concentrations of P and K were higher and the N:P and N:K ratios were lower in the fertilized than the non-fertilized saplings at the end of the experiment, 10 years after the NPK fertilizer had been applied (Supplementary Table S6). This finding suggests that N was more limiting than P and K over the course of the experiment and that the added N was depleted more quickly and could not be accumulated in the needles until the end of the experiment, as for P and K. These results thus support the idea that N is more limiting than other nutrients for trees that grow at the treeline (Körner, 2003) and is available mostly in a recalcitrant organic form. The positive effect of fertilization on stem growth, however, decreased to some extent when fertilization was combined with the presence of the shrub (Figure 2 and Supplementary Table S1A). *R. ferrugineum* thus profited more than *P. uncinata* from the higher availability of soil nutrients, probably because of its greater biomass (Epstein et al., 2000). We also found some support for hypothesis (5), that warming and fertilization would have an additive effect on tree performance; the trees were largest in the microplots where both treatments were combined. This finding suggests that the growth of the trees that were warmed but not fertilized was limited by nutrient availability, because the warmed trees grew more if they were also fertilized. This additive effect, however, only occurred when the shrub was not present, again suggesting that the presence of shrubs could buffer the effects of other factors.

The chemical composition of the *P. uncinata* needles differed greatly between the control and the warmed and/or fertilized trees (Figure 8). This shift could be explained by the ‘biogeochemical niche hypothesis,’ which states that the biogeochemical niche should determine the species-specific strategy of growth and uptake of resources when plants are exposed to changes in environmental conditions or suffer from competition with other species (Peñuelas et al., 2008). We would thus observe an expansion of the biogeochemical niche (an increase in stoichiometric differences between treatments), due to changes in the abiotic and biotic conditions (Urbina et al., 2017), suggesting a clear shift in the chemical properties of the needles, which possibly respond to changes in their physiology.

The ordinations in Figures 6, 8 clearly indicate that all experimental manipulations influenced the performance of *P. uncinata*, with important interactions amongst the treatments. The trees responded quickly at the start of the experiment (Grau et al., 2013), and these physiological responses and adaptations persisted, confirming that the trees growing at the Pyrenean treeline are persistently influenced by shrub-tree interactions and changes in temperature and nutrient availability. The mechanisms of these interactions, however, change over time, with facilitation playing an important role at the seedling stage and competition (and release from it) becoming more prominent at the sapling stage, as discussed above. This finding is in agreement with previous studies (e.g., Körner and Paulsen, 2004; Ameztegui and Coll, 2011; Liang et al., 2016) reporting that tree development at the treeline is highly sensitive to changes in both biotic and abiotic conditions.

## Conclusion

### Future Implications for Tree Development at the Pyrenean Treeline

The high sensitivity of *P. uncinata* seedlings (later saplings) to the experimental manipulations suggests that pines at the treeline will most likely respond to current and future changes in abiotic and biotic conditions. The ongoing expansion of shrub cover in this region (Molinillo et al., 1997; Roura-Pascual et al., 2005; Montané et al., 2007; Alados et al., 2011; Ninot et al., 2011; Garcia-Pausas et al., 2017) could favor an intensification of the interactions between shrubs and trees growing at the treeline. Our results indicate that both facilitation and competition may co-occur under such a scenario at the initial stage of tree development. The expansion of shrubs such as *R. ferrugineum* would favor the availability of safe sites and protect small trees from abiotic damage, especially in years with low snow cover, and enhance survival and tree establishment at the treeline, which could be especially relevant because of the statistically significant reduction of winter precipitation in recent decades (1959–2010) in the Pyrenean region and the increase in interannual variability of winter precipitation (López-Moreno, 2005; Cuadrat et al., 2013; Martín-Vide et al., 2017) that are likely to persist (IPCC, 2013). The shrubs, however, may compete with the trees for resources, especially light and nutrients, and thereby hamper their development. The balance between facilitation and competition between the shrubs and trees will thus strongly determine the establishment and development of new trees at the treeline.

Temperatures have also increased by +0.2°C per decade in recent decades in the Pyrenees, especially during spring and summer (Cuadrat et al., 2013; Martín-Vide et al., 2017). This trend is also predicted to continue (IPCC, 2013). Based on the results of our experiment, where we simulated an increase of ca. 2°C during the growing season, *P. uncinata* seedlings are expected to benefit from this ongoing thermal increase. Warmer conditions, together with the predictable increase in the availability of limiting nutrients such as N (Hobbie, 1996; Rustad et al., 2001), are also expected to favor the development of young *P. uncinata* individuals, based on the findings of our experiment. We predict, though, that nutrient availability will remain an important limiting factor in this system also in a future, warmer climate, as trees that were warmed but not fertilized grew less than those that were warmed and fertilized. The cover of *R. ferrugineum*, however, is expected to buffer these positive warming and fertilization effects on young individuals, suggesting that the interaction between abiotic and biotic factors may play a key role in future treeline dynamics, especially if shrub cover increases. We believe that our findings for dynamic shrub-tree interactions throughout the lifetime of young trees in treeline environments subjected to global change have important implications not only for treelines in the Pyrenees, but also for many other treelines around the world where shrubs and trees co-occur.

## Author Contributions

OG and JN conceived and designed the study. MA and OG wrote the first version of the manuscript. All authors actively contributed to revisions.

## Conflict of Interest Statement

The authors declare that the research was conducted in the absence of any commercial or financial relationships that could be construed as a potential conflict of interest.
