# Supplementary material for: Tree Sapling Responses to 10 Years of Experimental Manipulation of Temperature, Nutrient Availability, and Shrub Cover at the Pyrenean Treeline
Source: Front Plant Sci. 2019 Jan 8;9:1871. doi: 10.3389/fpls.2018.01871 (PMC6333114; doi:10.3389/fpls.2018.01871)
Supplement: Supplementary file 1 [file Table_1.DOCX]

Table S1. Statistical significance of the treatments for (A) stem height, (B) stem diameter, and (C) number of primary branches measured at the end of several growing seasons throughout the course of the experiment. “+” indicates a positive effect on the variable, and “-“ indicates a negative effect. Columns 2-6 in (A) show the significance from mixed models for each year separately; the last column shows the significance for the repeated-measures analysis for 2006-2016, which accounted for temporal autocorrelation. Treatments: S, shrub; T, increased temperature; F, fertilizer addition.

**(A) Stem height**

| **Treatment** | **2006** | **2008** | **2009** | **2015** | **2016** | **2006-2016 (Repeated measures)** |
| --- | --- | --- | --- | --- | --- | --- |
| **T** | n.s. | + (P =0.0037) | + (P = 0.0016) | + (P = 0.0001) | + (P = 0.0001) | + (P = 0.0003) |
| **F** | n.s. | n.s. | + (P = 0.0362) | + (P = 0.0355) | + (P = 0.0360) | + (P = 0.0192) |
| **S** | n.s. | n.s. | + (P = 0.0009) | + (P = 0.0051) | + (P = 0.0027) | n.s. |
| **T × F** | n.s. | n.s. | n.s. | n.s. | n.s. | n.s. |
| **T × S** | n.s. | n.s. | - (P = 0.0412) | - (P = 0.0000) | - (P = 0.0000) | - (P = 0.0032) |
| **F × S** | n.s. | n.s. | - (P = 0.0515) | - (P = 0.0755) | -( P = 0.0517) | - (P = 0.0725) |
| **F × T × S** | n.s. | n.s. | n.s. | n.s. | n.s. | n.s. |
| **Time** |  |  |  |  |  | + (P = 0.0000) |

**(B) Stem diameter**

| **Treatment** | **2009** | **2016** |
| --- | --- | --- |
| **T** | +(P = 0.0015) | + (P = 0.0036) |
| **F** | + (P = 0.0224) | n.s. |
| **S** | n.s. | n.s. |
| **T × F** | n.s. | n.s. |
| **T × S** | - (P = 0.0232) | - (P = 0.0001) |
| **F × S** | n.s. | n.s. |
| **F × T × S** | n.s. | n.s. |

**(C) Number of primary branches**

| **Treatment** | **2006** | **2008** | | **2016** |
| --- | --- | --- | --- | --- |
| **T** | n.s. | | - (P = 0.0884) | + (P = 0.0059) |
| **F** | n.s. | | + (P = 0.0059) | n.s. |
| **S** | n.s. | | + (P = 0.0010) | - (P = 0.00005) |
| **T × F** | n.s. | | n.s. | n.s. |
| **T × S** | n.s. | | n.s. | - (P = 0.00001) |
| **F × S** | n.s. | | n.s. | n.s. |
| **F × T × S** | n.s. | | n.s. | n.s. |
