# Supplementary material for: Tree Sapling Responses to 10 Years of Experimental Manipulation of Temperature, Nutrient Availability, and Shrub Cover at the Pyrenean Treeline
Source: Front Plant Sci. 2019 Jan 8;9:1871. doi: 10.3389/fpls.2018.01871 (PMC6333114; doi:10.3389/fpls.2018.01871)
Supplement: Supplementary file 2 [file Table_2.DOCX]

Table S2. Mean stem height (cm) (± standard deviation) in each treatment.

| **Treatment** | **2006** | **2007** | **2008** | **2009** | **2015** | **2016** |
| --- | --- | --- | --- | --- | --- | --- |
| **-S-T-F** | 7.25 ± 1.42 | 10.6 ± 1.72 | 20.26 ± 19.5 | 11.22 ± 2.23 | 26.09 ± 9.02 | 31.77 ± 9.71 |
| **-S-T+F** | 7.98 ± 1.40 | 10.51 ± 3.81 | 29.3 ± 20.29 | 11.85 ± 2.27 | 42.93 ± 13.81 | 49.61 ± 15.42 |
| **-S+T-F** | 7.93 ± 1.23 | 7.65 ± 5.12 | 25.59 ± 15.58 | 12.95 ± 2.44 | 58.59 ± 15.12 | 66.26 ± 14.89 |
| **-S+T+F** | 8.02 ± 1.56 | 10.5 ± 3.72 | 28.17 ± 12.96 | 15.61 ± 3.13 | 66.23 ± 22.26 | 74.75 ± 23.93 |
| **+S-T-F** | 6.80 ± 1.36 | 11.27 ± 1.64 | 14.88 ± 12.05 | 14.19 ± 1.93 | 37.96 ± 9.61 | 45.45 ± 11.42 |
| **+S-T+F** | 6.69 ± 1.21 | 11.48 ± 2.52 | 32.54 ± 18.55 | 13.76 ± 3.25 | 46.85 ± 11.08 | 54.28 ± 12.41 |
| **+S+T-F** | 6.20 ± 1.51 | 11.01 ± 2.9 | 28.68 ± 18.92 | 14.83 ± 2.99 | 44.93 ± 14.62 | 51.69 ± 12.32 |
| **+S+T+F** | 6.76 ± 1.64 | 10.46 ± 92 | 32.27 ± 17.78 | 14.39 ± 3.19 | 44.07 ± 14.26 | 50.84 ± 14.27 |
