# Supplementary material for: Tree Sapling Responses to 10 Years of Experimental Manipulation of Temperature, Nutrient Availability, and Shrub Cover at the Pyrenean Treeline
Source: Front Plant Sci. 2019 Jan 8;9:1871. doi: 10.3389/fpls.2018.01871 (PMC6333114; doi:10.3389/fpls.2018.01871)
Supplement: Supplementary file 3 [file Table_3.DOCX]

Table S3. Mean stem diameter (mm) (± standard deviation) in each treatment.

| **Treatment** | **2009** | **2016** |
| --- | --- | --- |
| **-S-T-F** | 0.36 ± 0.07 | 8.79 ± 3.53 |
| **-S-T+F** | 0.40 ± 0.06 | 13.09 ± 4.51 |
| **-S+T-F** | 0.43 ± 0.10 | 15.8 ± 4.44 |
| **-S+T+F** | 0.48 ± 0.10 | 17.72 ± 7.03 |
| **+S-T-F** | 0.35 ± 0.07 | 8.96 ± 2.78 |
| **+S-T+F** | 0.39 ± 0.06 | 11.5 ± 4.90 |
| **+S+T-F** | 0.37 ± 0.08 | 8.41 ± 3.86 |
| **+S+T+F** | 0.39 ± 0.07 | 8.8 ± 5.80 |
