# Supplementary material for: Tree Sapling Responses to 10 Years of Experimental Manipulation of Temperature, Nutrient Availability, and Shrub Cover at the Pyrenean Treeline
Source: Front Plant Sci. 2019 Jan 8;9:1871. doi: 10.3389/fpls.2018.01871 (PMC6333114; doi:10.3389/fpls.2018.01871)
Supplement: Supplementary file 4 [file Table_4.DOCX]

Table S4. Mean number of branches (± standard deviation) in each treatment*.*

| **Treatment** | **2006** | **2008** | **2016** |  |
| --- | --- | --- | --- | --- |
| **-S-T-F** | 2.21 ± 1.20 | 5.74 ± 2.90 | 12.65 ± 6.11 | |
| **-S-T+F** | 2.41 ± 0.88 | 6.7 ± 2.71 | 15.2 ± 5.12 | |
| **-S+T-F** | 2.13 ± 0.95 | 3.88 ± 1.27 | 18.27 ± 3.90 | |
| **-S+T+F** | 2.33 ± 1.14 | 5.71 ± 2.85 | 18.05 ± 5.15 | |
| **+S-T-F** | 1.71 ± 0.75 | 2.9 ± 0.94 | 10.5 ± 4.01 | |
| **+S-T+F** | 2.38 ± 1.50 | 4.76 ± 2.58 | 10.67 ± 4.07 | |
| **+S+T-F** | 1.71 ± 0.91 | 2.93 ± 1.34 | 8.47 ± 4.22 | |
| **+S+T+F** | 1.56 ± 0.70 | 4.17 ± 1.19 | 9.94 ± 5.53 | |
