# Supplementary material for: Tree Sapling Responses to 10 Years of Experimental Manipulation of Temperature, Nutrient Availability, and Shrub Cover at the Pyrenean Treeline
Source: Front Plant Sci. 2019 Jan 8;9:1871. doi: 10.3389/fpls.2018.01871 (PMC6333114; doi:10.3389/fpls.2018.01871)
Supplement: Supplementary file 5 [file Table_5.DOCX]

Table S5. Means (± standard deviations) for all morphological variables measured in 2016 in each treatment.

| **Treatment** | **Number of new secondary stems** | **Total biomass (g)** | **Biomass of new needles (g)** |
| --- | --- | --- | --- |
| **-S-T-F** | 34.24 ± 21.61 | 44.04 ± 31.18 | 11.3 ± 7.91 |
| **-S-T+F** | 46.35 ± 25.57 | 104.86 ± 76.67 | 24.69 ± 16.17 |
| **-S+T-F** | 45.8 ± 24.43 | 131. 54 ± 67.12 | 24.6 ± 12.2 |
| **-S+T+F** | 45.06 ± 24.21 | 143. 55 ± 104.59 | 31.05 ± 21.26 |
| **+S-T-F** | 19.92 ± 11.07 | 45.12 ± 26.95 | 11.91 ± 8.35 |
| **+S-T+F** | 28.33 ± 13.14 | 70.76 ± 55.83 | 16.45 ± 11.15 |
| **+S+T-F** | 14.95 ± 9.69 | 42.24 ± 37.85 | 7.51 ± 7.09 |
| **+S+T+F** | 20.94 ± 16.85 | 56.92 ± 73.43 | 11.43 ± 14.62 |
