# Supplementary material for: Tree Sapling Responses to 10 Years of Experimental Manipulation of Temperature, Nutrient Availability, and Shrub Cover at the Pyrenean Treeline
Source: Front Plant Sci. 2019 Jan 8;9:1871. doi: 10.3389/fpls.2018.01871 (PMC6333114; doi:10.3389/fpls.2018.01871)
Supplement: Supplementary file 6 [file Table_6.DOCX]

Table S6. Mean (± standard deviation) foliar C, nutrients, and isotopes in each treatment.

| **Treatment** | **C (g)** | **N (g)** | **∂C^13^** | **∂N^15^** |
| --- | --- | --- | --- | --- |
| **-S-T-F** | 1.80×10^-3^ ± 2.69×10^-5^ | 3.92×10^-5^ ± 9.82×10^-7^ | -27.24 ± 7.93×10^-2^ | -1.22 ± 1.23 |
| **-S-T+F** | 1.78×10^-3^ ± 2.22×10^-5^ | 4.14×10^-5^ ± 2.61×10^-6^ | -26.88 ± 3.64×10^-1^ | -0.44 ± 7.14×10^-1^ |
| **-S+T-F** | 1.81×10^-3^ ± 6.77×10^-5^ | 4.08×10^-5^ ± 2.36×10^-6^ | -27.07 ± 8.34×10^-1^ | -1.32 ± 2.63×10^-1^ |
| **-S+T+F** | 1.74×10^-3^ ± 1.16×10^-4^ | 4.11×10^-5^ ± 3.60×10^-6^ | -26.88 ± 3.08×10^-1^ | -0.99 ± 4.48×10^-1^ |
| **+S-T-F** | 1.79×10^-3^ ± 1.52×10^-5^ | 4.00×10^-5^ ± 2.18×10^-6^ | -26.99 ± 4.16×10^-1^ | -1.56 ± 1.55 |
| **+S-T+F** | 1.78×10^-3^ ± 1.44×10^-5^ | 3.93×10^-5^ ± 1.52×10^-6^ | -27.01 ± 3.66×10^-1^ | -2.07 ± 1.05 |
| **+S+T-F** | 1.78×10^-3^ ± 1.89×10^-5^ | 4.16×10^-5^ ± 4.10×10^-6^ | -27.11 ± 5.73×10^-1^ | -3.11 ± 7.71×10^-1^ |
| **+S+T+F** | 1.79×10^-3^ ± 1.77×10^-5^ | 4.18×10^-5^ ± 2.91×10^-6^ | -27.37 ± 6.67×10^-1^ | -1.94 ± 8.87×10^-1^ |

Table S6 (continued)

| **Treatment** | **P (%(p/p))** | **Mg (%(p/p))** | **K (%(p/p))** | **Ca (%(p/p))** | **Cr (mg/kg)** | **Mo (mg/kg)** |
| --- | --- | --- | --- | --- | --- | --- |
| **-S-T-F** | 0.109 ± 5.90×10^-3^ | 0.113 ± 1.71×10^-2^ | 0.420 ± 1.61×10^-1^ | 0.185 ± 5.00×10^-2^ | 3.80 ± 1.010 | 0.19 ± 0.097 |
| **-S-T+F** | 0.128 ± 8.22×10^-3^ | 0.108 ± 9.60×10^-3^ | 0.475 ± 4.40×10^-2^ | 0.158 ± 1.30×10^-2^ | 4.50 ± 2.238 | 0.16 ± 0.078 |
| **-S+T-F** | 0.119 ± 1.71×10^-2^ | 0.120 ± 2.71×10^-2^ | 0.425 ± 6.60×10^-2^ | 0.190 ± 2.90×10^-2^ | 5.45 ± 3.717 | 0.21 ± 0.105 |
| **-S+T+F** | 0.117 ± 1.74×10^-2^ | 0.108 ± 9.60×10^-3^ | 0.545 ± 1.05×10^-1^ | 0.185 ± 3.30×10^-2^ | 18.25 ± 29.84 | 0.26 ± 0.131 |
| **+S-T-F** | 0.113 ± 4.60×10^-3^ | 0.118 ± 9.00×10^-3^ | 0.415 ± 7.60×10^-2^ | 0.160 ± 2.20×10^-2^ | 3.10 ± 0.589 | 0.16 ± 0.079 |
| **+S-T+F** | 0.123 ± 6.90×10^-3^ | 0.105 ± 5.80×10^-3^ | 0.558 ± 4.30×10^-2^ | 0.155 ± 1.00×10^-2^ | 6.90 ± 2.445 | 0.18 ± 0.090 |
| **+S+T-F** | 0.115 ± 1.42×10^-2^ | 0.118 ± 1.50×10^-2^ | 0.478 ± 2.10×10^-2^ | 0.173 ± 2.20×10^-2^ | 4.45 ± 3.754 | 0.37 ± 0.187 |
| **+S+T+F** | 0.133 ± 1.68×10^-2^ | 0.110 ± 3.27×10^-2^ | 0.635 ± 1.17×10^-1^ | 0.190 ± 8.30×10^-2^ | 3.98 ± 2.147 | 0.43 ± 0.213 |

Table S6 (continued)

| **Treatment** | **Mn (mg/kg)** | **Fe (mg/kg)** | **Ni (mg/kg)** | **Cu (mg/kg)** | **Zn (mg/kg)** | **Sr (mg/kg)** |
| --- | --- | --- | --- | --- | --- | --- |
| **-S-T-F** | 123.75 ± 54.537 | 66.75 ± 5.058 | 2.950 ± 0.592 | 2.45 ± 0.208 | 21.80 ± 1.467 | 3.99 ± 0.211 |
| **-S-T+F** | 93.50 ± 39.501 | 79.50 ± 17.330 | 3.000 ± 0.898 | 3.08 ± 0.299 | 22.58 ± 2.480 | 2.56 ± 0.615 |
| **-S+T-F** | 164.75 ± 35.312 | 80.50 ± 19.227 | 3.875 ± 1.765 | 3.02 ± 0.395 | 25.95 ± 0.911 | 3.01 ± 0.873 |
| **-S+T+F** | 131.00 ± 38.497 | 176.00 ± 226.013 | 3.350 ± 0.695 | 2.90 ± 0.271 | 24.70 ± 3.244 | 2.92 ± 1.827 |
| **+S-T-F** | 64.50 ± 14.480 | 69.00 ± 6.218 | 2.275 ± 0.330 | 2.68 ± 0.330 | 23.05 ± 2.340 | 3.68 ± 0.857 |
| **+S-T+F** | 75.25 ± 26.094 | 86.50 ± 21.977 | 3.525 ± 1.050 | 2.83 ± 0.263 | 24.83 ± 3.207 | 2.24 ± 0.328 |
| **+S+T-F** | 107.25 ± 25.786 | 66.25 ± 26.650 | 2.925 ± 2.341 | 2.95 ± 0.777 | 29.48 ± 5.965 | 5.04 ± 2.034 |
| **+S+T+F** | 108.25 ± 17.914 | 63.50 ± 7.594 | 2.975 ± 1.167 | 3.10 ± 0.469 | 29.33 ± 6.724 | 5.83 ± 7.368 |

Table S6 (continued)

| **Treatment** | **Na (%(p/p))** | **S (%(p/p))** | **V (mg/kg)** | **As (mg/kg)** | **Cd (mg/kg)** | **Pb (mg/kg)** |
| --- | --- | --- | --- | --- | --- | --- |
| **-S-T-F** | <200 | <0.4 | <1 | <0.1 | <0.1 | <0.1 |
| **-S-T+F** | <200 | <0.4 | <1 | <0.1 | <0.1 | <0.1 |
| **-S+T-F** | <200 | <0.4 | <1 | <0.1 | <0.1 | <0.1 |
| **-S+T+F** | <200 | <0.4 | <1 | <0.1 | <0.1 | <0.1 |
| **+S-T-F** | <200 | <0.4 | <1 | <0.1 | <0.1 | <0.1 |
| **+S-T+F** | <200 | <0.4 | <1 | <0.1 | <0.1 | <0.1 |
| **+S+T-F** | <200 | <0.4 | <1 | <0.1 | <0.1 | <0.1 |
| **+S+T+F** | <200 | <0.4 | <1 | <0.1 | <0.1 | <0.1 |
