# Supplementary material for: Tree Sapling Responses to 10 Years of Experimental Manipulation of Temperature, Nutrient Availability, and Shrub Cover at the Pyrenean Treeline
Source: Front Plant Sci. 2019 Jan 8;9:1871. doi: 10.3389/fpls.2018.01871 (PMC6333114; doi:10.3389/fpls.2018.01871)
Supplement: Supplementary file 7 [file Table_7.DOCX]

Table S7. Statistical significance of the treatments for variables measured in 2016. “+” indicates a positive effect on the variable, and “-” indicates a negative effect.

| **Treatment** | **Total biomass** | **Biomass of new secondary stems** | **Number of new secondary stems** | **Biomass of new needles** |
| --- | --- | --- | --- | --- |
| **T** | + (P = 0.0261) | + (P = 0.0271) | n.s. | + (P = 0.0792) |
| **F** | n.s. | n.s. | n.s. | n.s. |
| **S** | n.s. | n.s. | - (P = 8.16 x 10^-7^) | n.s. |
| **T × F** | n.s. | n.s. | n.s. | n.s. |
| **T × S** | - (P = 0.0004) | - (P =0.0008) | - (P = 0.0146) | - (P = 0.0018) |
| **F × S** | n.s. | n.s. | n.s. | n.s. |
| **F × T × S** | n.s. | n.s. | n.s. | n.s. |
