# Supplementary material for: Tree Sapling Responses to 10 Years of Experimental Manipulation of Temperature, Nutrient Availability, and Shrub Cover at the Pyrenean Treeline
Source: Front Plant Sci. 2019 Jan 8;9:1871. doi: 10.3389/fpls.2018.01871 (PMC6333114; doi:10.3389/fpls.2018.01871)
Supplement: Supplementary file 8 [file Table_8.DOCX]

Table S8. Statistical significance of the treatments for foliar chemical ratios. “+” indicates a positive effect on the variable, and “-” indicates a negative effect. The C:N:P:K ratios that were not significant for at least one treatment are not shown.

| **Treatment** | **N:P** | **C:K** | **N:K** |
| --- | --- | --- | --- |
| **T** | n.s. | n.s. | n.s. |
| **F** | - (P = 0.0694) | - (P = 0.0015) | - (P = 0.0036) |
| **S** | n.s. | - (P = 0.0653) | n.s. |
| **T × F** | n.s. | n.s. | n.s. |
| **T × S** | n.s. | n.s. | n.s. |
| **F × S** | n.s. | n.s. | n.s. |
| **F × T × S** | n.s. | n.s. | n.s. |
