# Supplementary material for: Tree Sapling Responses to 10 Years of Experimental Manipulation of Temperature, Nutrient Availability, and Shrub Cover at the Pyrenean Treeline
Source: Front Plant Sci. 2019 Jan 8;9:1871. doi: 10.3389/fpls.2018.01871 (PMC6333114; doi:10.3389/fpls.2018.01871)
Supplement: Supplementary file 9 [file Table_9.docx]

Table S9. Statistical significance of the treatments for (A) foliar nutrient and ∂N^15^ and ∂C^13^ concentrations and (B) total nutrient contents. “+” indicates a positive effect on the variable, and “-” indicates a negative effect.

**(A) Nutrient concentration (g of each chemical element per g of needle)**

| **Treatment** | **C** | **N** | **P** | **K** | **Mn** | **Cu** | **Zn** | **Sr** | **∂N^15^** | **∂C^13^** |
| --- | --- | --- | --- | --- | --- | --- | --- | --- | --- | --- |
| **T** | n.s. | n.s. | n.s. | + (P = 0.0716) | + (P = 0.0069) | + (P = 0.0724) | + (P = 0.0063) | n.s. | n.s. | n.s. |
| **F** | n.s. | n.s. | + (P = 0.0260) | + (P = 0.0008) | - (P = 0.0662) | + (P = 0.0535) | n.s. | n.s. | n.s. | n.s. |
| **S** | n.s. | n.s. | n.s. | + (P = 0.0595) | - (P = 0.0014) | n.s. | + (P = 0.0333) | n.s. | n.s. | n.s. |
| **T × F** | n.s. | n.s. | n.s. | n.s. | n.s. | n.s. | n.s. | n.s. | n.s. | n.s. |
| **T × S** | n.s. | n.s. | n.s. | n.s. | n.s. | n.s. | n.s. | + (P = 0.0698) | - (P = 0.0260) | n.s. |
| **F × S** | n.s. | n.s. | n.s. | n.s. | n.s. | n.s. | n.s. | n.s. | - (P = 0.0443) | n.s. |
| **F × T × S** | n.s. | n.s. | n.s. | n.s. | n.s. | n.s. | n.s. | n.s. | +( P = 0.0217) | n.s. |

**(B) Total content of chemical elements in saplings (g in total)**

| **Treatment** | **C** | **N** | **P** | **K** | **Mn** | **Cu** | **Zn** | **Sr** |
| --- | --- | --- | --- | --- | --- | --- | --- | --- |
| **T** | n.s. | n.s. | + (P = 0.0629) | n.s. | + (P = 0.0010) | + (P = 0.0532) | + (P = 0.0731) | n.s. |
| **F** | n.s. | n.s. | n.s. | n.s. | n.s. | + (P = 0.0635) | n.s. | n.s. |
| **S** | n.s. | n.s. | n.s. | n.s. | n.s. | n.s. | n.s. | - (P = 0.0811) |
| **T × F** | n.s. | n.s. | n.s. | n.s. | n.s. | n.s. | n.s. | n.s. |
| **T × S** | - (P = 0.0024) | - (P = 0.0028) | - (P = 0.0437) | - (P = 0.0042) | - (P = 0.0041) | - (P = 0.0033) | - (P =0.0005 ) | n.s. |
| **F × S** | n.s. | n.s. | n.s. | n.s. | n.s. | - (P = 0.0723) | n.s. | n.s. |
| **F × T × S** | n.s. | n.s. | n.s. | n.s. | n.s. | n.s. | n.s. | n.s. |
