# Supplementary figures and images for: Tree Sapling Responses to 10 Years of Experimental Manipulation of Temperature, Nutrient Availability, and Shrub Cover at the Pyrenean Treeline
Source: Front Plant Sci. 2019 Jan 8;9:1871. doi: 10.3389/fpls.2018.01871 (PMC6333114; doi:10.3389/fpls.2018.01871)

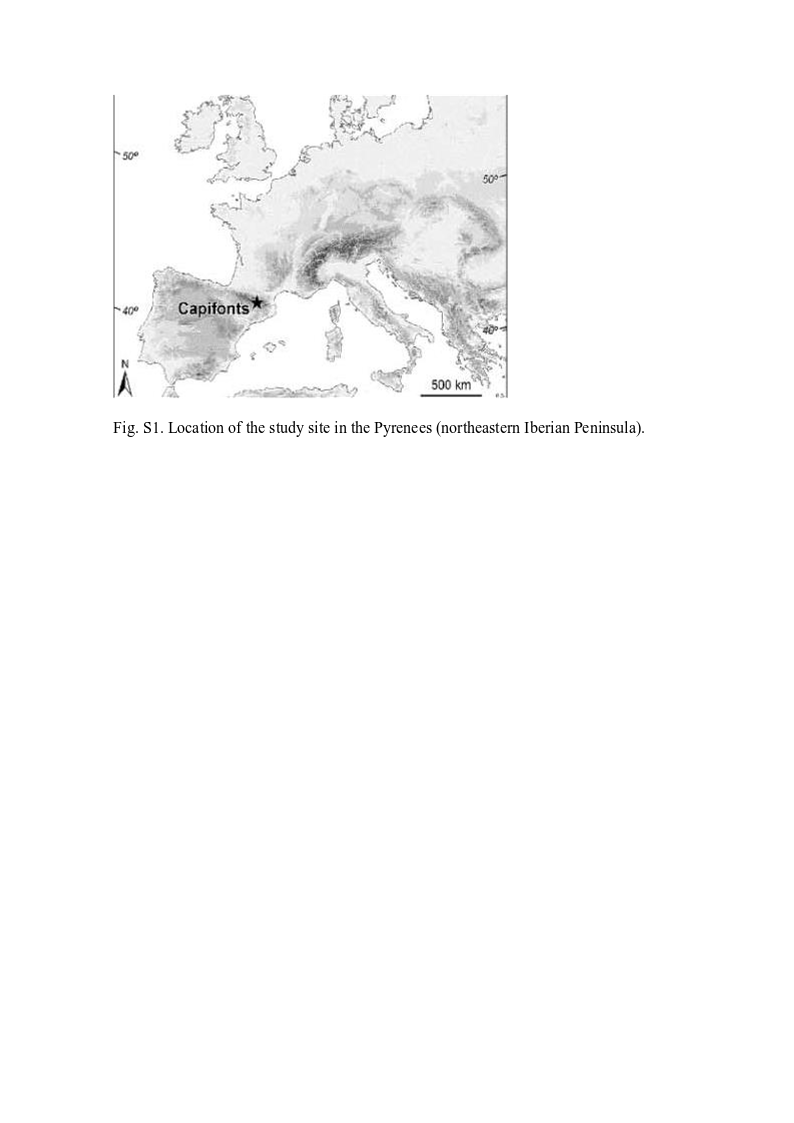

Supplement: Supplementary file 10 [file Image_1.PNG]

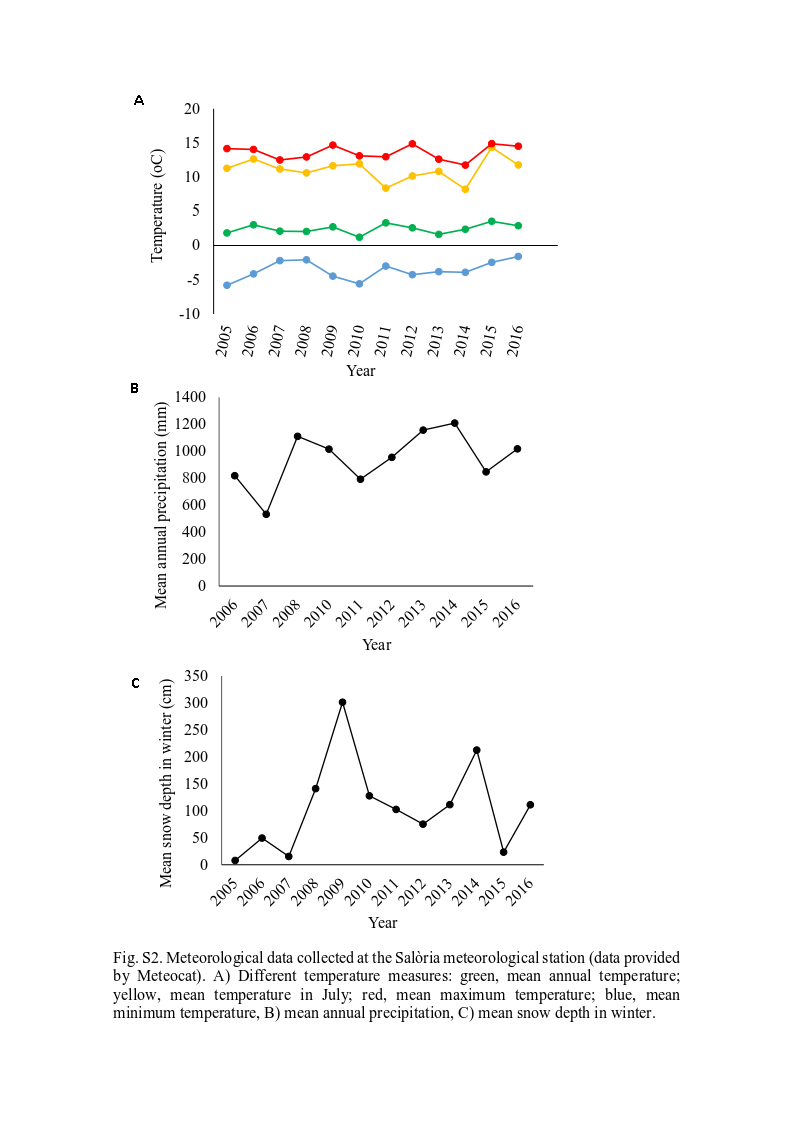

Supplement: Supplementary file 11 [file Image_2.PNG]

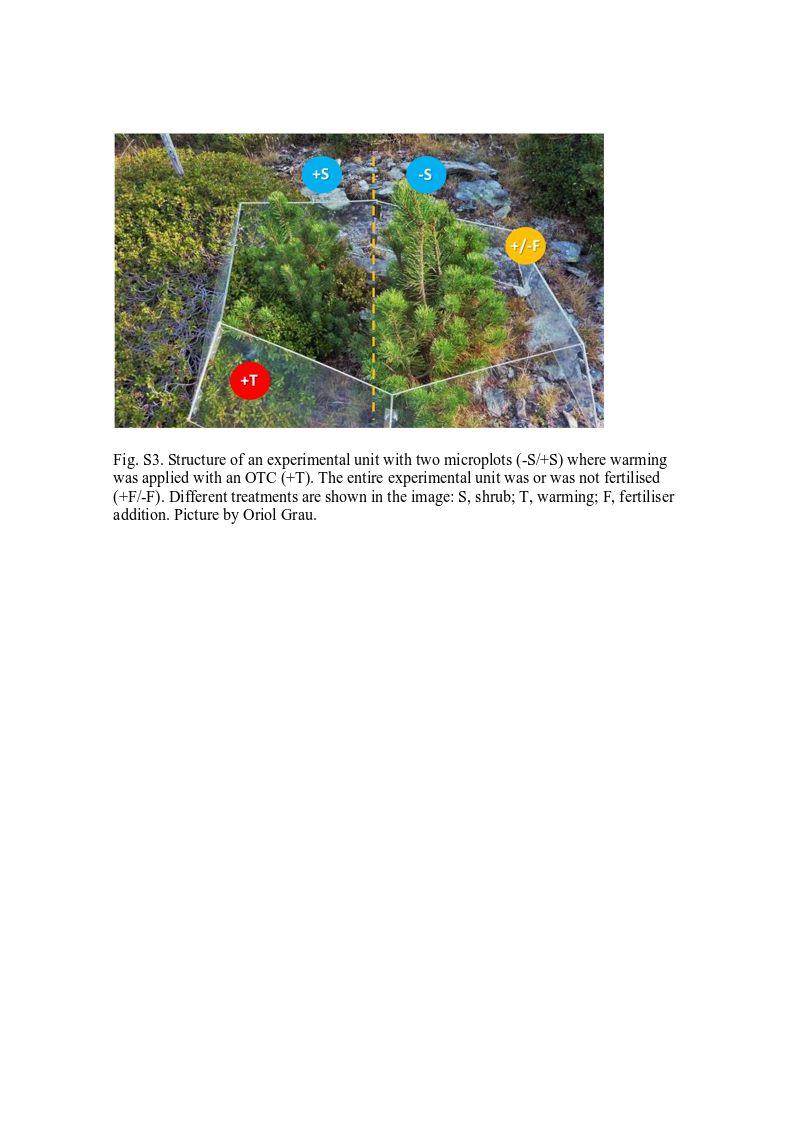

Supplement: Supplementary file 12 [file Image_3.PNG]

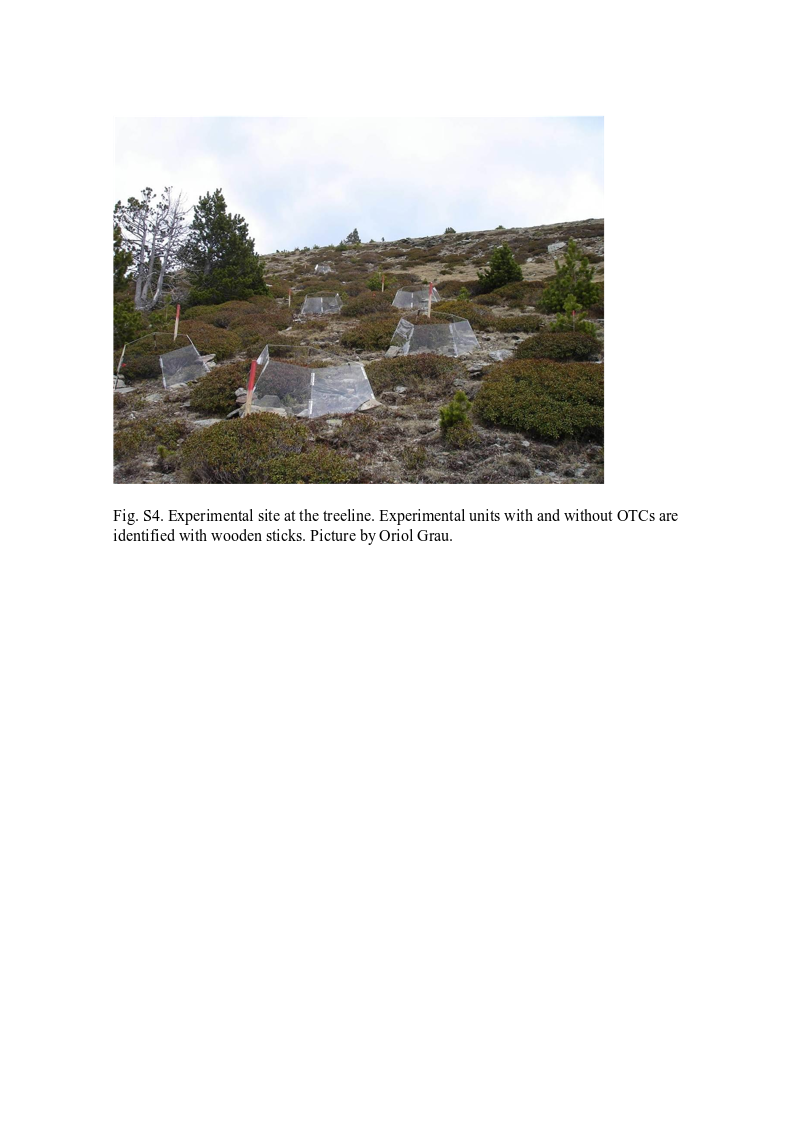

Supplement: Supplementary file 13 [file Image_4.PNG]
